# The forensic aspects of suicide and neurotrophin factors: a research study

**DOI:** 10.3389/fphar.2024.1392832

**Published:** 2024-08-07

**Authors:** Stefania De Simone, Letizia Alfieri, Maria Antonella Bosco, Santina Cantatore, Michele Carpinteri, Luigi Cipolloni, Margherita Neri

**Affiliations:** ^1^ Department of Clinical and Experimental Medicine, Section of Legal Medicine, University of Foggia, Foggia, Italy; ^2^ Department of Medical Sciences, Section of Legal Medicine University of Ferrara, Ferrara, Italy; ^3^ Department of Biomedical, Metabolic and Neural Sciences, Institute of Legal Medicine, University of Modena and Reggio Emilia, Modena, Italy

**Keywords:** suicide, neurotrophic factor, immunohistochemistry, BDNF, GDNF

## Abstract

**Introduction:** Suicide represents a significant public health problem whose neurobiology is not yet fully understood. In many cases, suicidal behavior and psychiatric spectrum disorders are linked, in particular, to major depression. An emerging pathophysiological hypothesis underlines the role of neurotrophic factors, proteins involved in neurogenesis, in synaptic plasticity in response to stressors. Our research aims to evaluate the degree of expression of brain neurotrophic factor (BDNF) in brain areas involved in depressive disorder in suicidal subjects. Furthermore, we want to evaluate the expression of glial cell line-derived neurotrophic factor (GDNF) in suicidal subjects.

**Methods:** We selected twenty confirmed cases of suicide among subjects with a clinical history of depressive pathology and possible psychopharmacological treatment, compared to ten controls of individuals who died of non-suicidal causes. For all selected cases and controls, immunohistochemical investigations were performed using a panel of antibodies against the BDNF and GDNF antigens on samples from the various brain areas.

**Results and discussion:** The results show that BDNF was under-expressed in the cerebral parenchyma of subjects who died by suicide compared to controls, while there was an overexpression of GDNF in suicide victims, these data could be useful for a clinical application as potential markers for suicidal risk, to assess the severity of depression and development of specific pharmacological therapies for depression.

## 1 Introduction

According to the WHO, every year, about 700,000 people end their lives through suicide, making it the fourth leading cause of death in young people (datadot, 2024). According to estimates, for every suicide carried out, there are between 10 and 40 suicide attempts (Krug et al., 2002; Chang et al., 2011). Suicide turns out to be a real social problem, as it cannot leave public opinion indifferent. According to ISTAT (Italian National Institute of Statistics) data from 2016, 3780 committed suicides in Italy, 78.8% of whom were male. The death rate from suicide is 11.8 per 100,000 population for men and 3.0 per 100,000 population for women (Vichi et al., 2019). In Western countries, the suicide rate among men is three to four times higher than among women. This difference is likely to be the case because men use reliably more lethal means of taking their own lives. This difference is even more marked in men aged 65 years and older, who are likely to be ten times more than women (Sue et al., 2016). In the Eastern Mediterranean countries, on the other hand, female and male suicide rates are almost identical. In many countries, suicide rates appear to be higher in middle age or old age. The absolute number of suicides, however, is highest in the age group between 15 and 29 years old (Pitman et al., 2012; Yip et al., 2012). Suicide is also related to age, with young people under 25 and older adults interested the most in the risk of suicide, although it proportionally increases with age (OECD, 2012). In 2018, the ISTAT estimated that 3,820 people committed suicide, with the highest incidence in males between 35 and 64 years old. The main methods used to commit suicide are hanging, pesticide poisoning, and the use of firearms. In a survey (Ajdacic-Gross et al., 2008) carried out in 56 countries, hanging was by far the most common method used by 53% of male suicides and 39% of female suicides (O Connor et al., 2011; Suicide in the Province, 2022). Suicide presents numerous risk factors (Beghi et al., 2021; Risk and Protective Factors, 2023), such as individuals (previous suicide attempts, chronic psychiatric and physical pathologies, drug addiction), relationships (history of family suicide, isolation, bullying), community (violence, discrimination), and social-linked (spectacularization by the media, access to lethal means). One of the most important risk factors is the presence of psychiatric pathology, primarily major depression, followed by anxiety disorder, bipolar disorder, and drug addiction (Beghi et al., 2021). Also, genetics can influence the risk of committing a self-suppressive act (Brent and Melhem, 2008). Among psychiatric pathologies, it is clear that patients suffering from major depression present a significant risk of suicide or attempted suicide (Conejero et al., 2018; Fusar-Poli et al., 2021). Identifying risk factors can recognize a part of the population at high risk of committing extreme acts (Granier and Boulenger, 2002).

The neurobiology underlying suicide is exceptionally complex, not being the result of a single pathophysiological entity but of a multiplicity of behavioral, socio-environmental, and psychological causal factors that act together simultaneously. According to the literature, suicide follows the “diathesis-stress” model: a behavioral disorder derives from a genetic predisposition that makes one more vulnerable to stress, usually caused by life events (Mann, 2003; Van Heeringen and Mann, 2014; Zeigler-Hill and Shackelford, 2019). In particular, traumatic life events and stress acts as triggers for suicidal behavior (Dwivedi, 2012). Furthermore, the interaction of numerous biological systems is involved (Girardi and Pompili, 2015), including the neurotrophic system. Indeed, the pathogenesis of depression and suicidal behavior involves changes in neuronal plasticity (Garcia, 2002), reducing the brain’s ability to adapt.

Neurotrophins are growth factors that play a crucial role in regulating structural and synaptic plasticity, maintaining neuronal functions, and modulating neurotransmission (Thoenen, 1995). Neurotrophins are essential for the survival, growth, and regeneration of different neuronal populations belonging to the central and peripheral nervous systems in the fetal and adult brain (Allen et al., 2013). The first neurotrophin discovered in the 1950s was Nerve Growth Factor (NGF) (Levi-Montalcini, 1987). Neurotrophin is transported from the production site to the nerve terminal via axonal retrograde flow during development. Neurons that present this retrograde flow survive; otherwise, they degenerate (“neurotrophic hypothesis”) (Hamburger et al., 1981; Ginty and Segal, 2002). There are five neurotrophic factors expressed in mammals: Brain-Derived Neurotrophic Factor (BDNF), Neurotrophins 3 and 4 (NT-3 and NT-4), Glial Derived Neurotrophic Factor (GDNF) and Ciliar Neurotrophic Factor (CNTF). Neurotrophins are initially synthesized as pro-neurotrophins, and then through proteolytic processes, they are converted into mature neurotrophins that interact with two classes of receptors (p75NRT and Trk) to act (Ibáñez and Simi, 2012; Neurotrophic, 2017). Neurotrophins also have numerous actions outside the nervous system, such as immune (Thorpe and Perez Polo, 1987; Otten et al., 1989; Donovan et al., 2000) and hematopoietic function (Thorpe et al., 1987). They also control some neuroendocrine functions (for example, the development of the female rat’s hypothalamus) (Ojeda et al., 1991) and are involved in the transmission of pain (Skaper, 2017). Furthermore, these molecules can increase cholinergic function and protect against neurodegeneration (Hock et al., 2000; Hoxha et al., 2018), hypothesizing a role in Alzheimer’s disease (Hefti, 1986). Similarly, the GDNF can be involved in Parkinson’s disease, as it acts on dopaminergic neurons (Yasuda and Mochizuki, 2010). Therefore, a pathological alteration of neurotrophic factors could determine defects in neural maintenance and regeneration, possible structural anomalies in the brain, and a reduction in neural plasticity, thus compromising the individual’s ability to adapt in critical situations.

BDNF (“Brain-Derived Neurotrophic Factor”) is the most widespread neurotrophin in the brain of mammals, both in adults and in the developing phase (Anisman et al., 2012). It is involved in neurogenesis, the development of neurons, neuronal homeostasis, the structural integrity and maintenance of neuronal plasticity in the adult brain (Altar et al., 1997; Bartrup et al., 1997; Edelmann et al., 2014; Panja and Bramham, 2014), synaptic connection, in the mechanisms that regulate learning and memory (Kang and Schuman, 1995), drug addiction, and in stress adaptation mechanisms. Numerous exogenous and endogenous stimuli (such as stress, physical activity, diet, and brain injury) regulate BDNF expression (Aid et al., 2007; Pruunsild et al., 2007). Several neuropathologies cause a reduction in BDNF protein levels and serum in patients’ brains (Autry and Monteggia, 2012; Borba et al., 2016). Unfortunately, it is unclear whether serum BDNF levels reflect brain levels, as studies contradict each other (Sartorius et al., 2009; Klein et al., 2011). However, serum BDNF levels should be higher than those in plasma and cerebrospinal fluid (Radka et al., 1996; Serra-Millàs, 2016). According to some clinical studies that examined the blood levels of BDNF *in vivo*, the BDNF can cross the blood-brain barrier in both directions via a high-capacity saturable transport system, which determines an early influx and rapid in the brain (Pan et al., 1998). BDNF concentrations can be measured in serum, plasma, or whole blood and appear highly correlated to those in cerebrospinal fluid. Therefore, BDNF levels in the blood could be related to the concentration of the same neurotrophin at the level of the cerebral cortex (Klein et al., 2011). The blood quantification of BDNF *in vivo* could be a marker of neuronal plasticity (Shimizu et al., 2003). Furthermore, BDNF also plays an essential role in neuroinflammation and aging, identifying a role in degenerative diseases such as Parkinson’s and Alzheimer’s (Lima et al., 2019). In humans, BDNF is considered a promising biomarker for various psychiatric pathologies (Chen et al., 2017). In some animal models, mice heterozygous for the BDNF gene showed increased aggression and anxiety, significant weight gain, and memory impairment, suggesting that its depletion could be associated with the development of some psychiatric symptoms (Lindholm and Castracn, 2014). This hypothesis also seems supported by the fact that there is an increase in BDNF levels after treatment of psychiatric pathologies (Nuernberg et al., 2016; Castrén and Monteggia, 2021). Patients with major depression show an appreciable reduction in serum levels of BDNF (Shimizu et al., 2003), probably due to a reduction of the protein in the brain (Karege et al., 2005a). There is also a single nucleotide polymorphism of the BDNF gene (Val66Met) that is associated with the severity of depressive symptoms and memory deficits (Youssef et al., 2018; Zhao et al., 2018), as well as a higher risk of suicide attempts (Zai et al., 2012). Typically, treatment with antidepressant drugs increases BDNF levels in serum and plasma (Molendijk et al., 2011). Some post-mortem studies have shown that treatment-resistant patients had significantly low levels of BDNF, especially in areas such as the hippocampus, which otherwise is generally rich (Colla et al., 2007; Brunoni et al., 2014). Other studies have demonstrated reductions in levels of BDNF mRNA and its protein from post-mortem brain samples of depressed patients, in particular in the hippocampus and amygdala (Guilloux et al., 2012; Ray et al., 2014). The reduction of serum BDNF levels has also been ascertained in other psychiatric pathologies, such as schizophrenia (Ray et al., 2014), autism (Katoh Semba et al., 2007), and bipolar disorder (Fernandes et al., 2015). Furthermore, in animal models, BDNF levels in the prefrontal cortex and hippocampus were increased after pharmacological treatment with mood stabilizers (Yasuda et al., 2009; Jornada et al., 2010). In the literature, there are numerous studies carried out on the brains of suicidal victims, which observe a reduction in the levels of BDNF protein and its receptor in the hippocampus (Karege et al., 2005a; University, 2021) and the prefrontal cortex (Zheng et al., 2016; Misztak et al., 2020), as well as in its mRNA (Hamburger et al., 1981). Thus, BDNF levels could represent an interesting biological marker of suicidal behavior.

Glial cell line-derived neurotrophic factor (GDNF) is a neurotrophin expressed primarily during neuronal development and differentiation. In the adult, its expression decreases and remains limited to some regions, such as the cortex, the hippocampus, the substantia nigra, and the striatum nucleus (Duarte Azevedo et al., 2020). GDNF promotes the differentiation of dopaminergic (Christophersen et al., 2007) and serotonergic (Ducray et al., 2006) neurons. Furthermore, one of its most important roles is the protection of neurons (Hochstrasser et al., 2011; Uzdensky et al., 2013; Jaumotte and Zigmond, 2014) from oxidative stress and inflammation (Pascual et al., 2008). GDNF levels appear to be reduced in patients who have Alzheimer’s disease, leading to the hypothesis of its use as a biomarker for the pathology [ (Sharif et al., 2021; Kimhy et al., 2015)]. Although there are not many studies in the literature relating to the involvement of GDNF in the suicidal phenomenon, there is evidence linking this neurotrophin to the onset of mood disorders. In fact, according to some studies, there are lower serum GDNF levels in patients suffering from depression, with a subsequent increase after pharmacological treatment (Lin and Tseng, 2015; Wang et al., 2023). In a post-mortem study, an increase in GDNF was observed in the parietal cortex (site of emotion regulation (Anderson et al., 2004)) of patients with depressive disorder (Michel et al., 2008), while in other studies, a reduction in the expression of its mRNA was measured (Otsuki et al., 2008). In the literature, GDNF levels increase following ischemic or inflammatory damage (Wei et al., 2000; Michel et al., 2007) as neuronal resilience and plasticity compromise, hypothesizing that the increase in GDNF is an adaptive and compensatory response to neuronal damage (Chao and Lee, 1999; Tokumine et al., 2003).

Currently, many studies on the possible link between neurotrophins and suicide are carried out *in vivo* or plasma (Kim et al., 2007; Salas Magana et al., 2017; Sonal and Raghavan, 2018). Further interesting data could come from post-mortem studies carried out directly on the brains of suicidal subjects. An essential element is that many of these studies focus on the link between neurotrophins and neuro-psychiatric pathology. However, not all subjects who commit suicide suffer from a diagnosed psychiatric pathology. A recent literature review (De Simone et al., 2022) shows a paucity of experimental work on BDNF and GDNF. It shows that the altered regulation of BDNF, such as the Val66Met polymorphism, can favor the onset of psychiatric disorders linked to stress (Felmingham et al., 2013; Zhao et al., 2018; Miao et al., 2020), leading to an increase in suicidal risk according to some works (Zai et al., 2012; Paska et al., 2013), or no correlation with suicide, according to another study (Ratta-apha et al., 2013). Some of the studies examining the level of BDNF in post-mortem brain samples showed reduced values compared to controls, regardless of psychiatric diagnosis, in the prefrontal cortex and hippocampus (Dwivedi et al., 2003; Karege et al., 2005b; Ducray et al., 2006; Hochstrasser et al., 2011), as well as in the amygdala (Ray et al., 2014) and the anterior cingulate cortex (Tripp et al., 2012) of subjects suffering from major depressive disorder. Schneider et al. (2015) also evaluated subjects suffering from depression, detecting increased methylation of the BDNF promoter in the frontal cortex, in agreement with other studies (Keller et al., 2010; Kang et al., 2013). Another interesting finding, supported by further evidence (Pan et al., 1998; Klein et al., 2011), is that the brain level of BDNF was higher in suicidal subjects suffering from major depression on pharmacological therapy compared to non-suicidal controls (Gadad et al., 2021). As regards GDNF, in the study by Michel et al. (Michel et al., 2007), it was not possible to demonstrate a statistically significant increase in the levels of this neurotrophin in different brain areas of depressed patients taking antidepressant drug therapy.

The results of this systematic review partially support the hypothesis that a lower level of neurotrophins is connected to an increased risk of suicide. The identification of a suicide biomarker remains a challenge for the scientific and forensic community. The objective of the present study is to analyze the correlation between suicide and the degree of expression of BDNF and GDNF on autopsy samples in specific brain areas. These neurotrophic factors could have an important role both in the prevention of suicidal events in the population at high risk for anti-conservative behavior, allowing early action to limit this risk preventively, and in the search for a new potential pharmacological target. In the literature, there are no valuable markers for the identification of suicidal risk, so BDNF and GDNF could be promising for identifying suicidal risk in people with well-defined risk factors.

## 2 Materials and methods

### 2.1 Sample selection

We conducted a retrospective study based on a series of judicial autopsies performed at the Forensic Medicine of the University of Foggia and the University of Ferrara.

The sample consists of twenty subjects who died following suicide between November 2020 and March 2023 (Table 1). The autopsies were performed 36–48 after the death and we excluded from the study corpses in an advanced stage of decomposition, corpses testing positive for common substances of abuse, and subjects suffering from neurodegenerative diseases, Alzheimer’s, and Parkinson’s disease. For subjects who used drugs, only benzodiazepines in three cases, we dosed the active ingredients, ensuring that they were at therapeutic doses (between 2 and 5 mg/day). We selected ten case controls, chosen among subjects who died of natural or only chest traumatic causes, without a history of psychiatric pathology or drug addiction, and who were negative for toxicological analyses.

**TABLE 1:** The table shows the main information on the selected cases.

N°	Sex	Age	Manner	Psychiatric history	Previous suicide attempts	Pharmacological therapy
1	M	40	Gunshot injury	Pathological gambling	No	No
2	M	36	Hanging	Reported job loss	No	No
3	F	39	Precipitation	Anorexia nervosa and bipolar disorder	No	No^a^
4	M	40	Precipitation	Substance use disorder	No	No
5	M	21	Precipitation	Alcoholism	No	
6	M	56	Hanging	Major depressive disorder	No	No
7	M	37	Hanging	Substance use disorder	No	No
8	M	24	Precipitation	Autism spectrum disorder	No	No
9	M	65	Hanging	Major depressive disorder	No	No
10	M	35	Hanging	Substance use disorder Convicted	No	No^a^
11	M	42	Hanging	Not diagnosed Convicted	No	No
12	M	46	Hanging	Convicted	Yes	No
13	M	35	Hanging	Adjustment disorder Convicted	Yes	No^a^
14	M	89	Use of a sharp weapon	Not diagnosed	Yes	No
15	M	36	Hanging	Convicted	No	No
16	F	22	Hanging	Self-harm History of childhood abuse	No	No
17	M	58	Hanging	Major depressive disorder	No	No
18	M	85	Use of a sharp weapon	Previous mournful events reported	No	No
19	M	62	Hanging	Major depressive disorder	No	No
20	M	84	Hanging	Major depressive disorder	No	No

^a^ Indicates subjects on pharmacological therapy with drugs (benzodiazepines) in doses within the therapeutic range**.**

In our sample, six individuals did not have a psychiatric diagnosis. However, they presented particularly critical and stressful recent events (e.g., bereavement, loss of job, imprisonment). The remaining fourteen cases, however, had a known psychiatric pathology; of these, six were affected by mood disorders (five from major depressive disorder, one from bipolar disorder), eight from other types of diagnosed psychiatric pathology (pathological gambling, substance use disorder, problematic use of alcohol, autism spectrum disorder, self-harm, adjustment disorder). The characteristics of these disorders are summarized in Table 2 based on the Diagnostic and Statistical Manual of Mental Disorders, 5th Edition (DSM-5). In all cases, we performed toxicological analyses on biological fluids to test the primary substances of abuse and the most common pharmacological active ingredients.

**TABLE 2:** The table shows the most relevant features of psychiatric disorders diagnosed in the selected cases, based on the Diagnostic and Statistical Manual of Mental Disorders, 5th Edition (DSM-5).

Disorder	No of subject affected	Definition based on DSM-V
Major depressive disorder	5	An individual must have five of the following-mentioned symptoms: ersistently low or depressed mood, anhedonia or decreased interest in pleasurable activities, feelings of guilt or worthlessness, lack of energy, poor concentration, appetite changes, psychomotor retardation or agitation, sleep disturbances, or suicidal thoughts. One must be a depressed mood or anhedonia causing social or occupational impairment
Bipolar disorder	1	Chronically occurring episodes of mania or hypomania alternating with depression. A manic episode is defined as a distinct period of persistently elevated or irritable mood with increased activity or energy lasting for at least seven consecutive days or requiring hospitalization. A hypomanic episode is defined as a distinct period of persistently elevated or irritable mood with increased activity or energy lasting for at least four consecutive days
Pathological gambling	1	Persistent, recurrent maladaptive patterns of gambling behavior, associated with impaired functioning, reduced quality of life, and high rates of bankruptcy, divorce, and incarceration
Substance use disorder	3	Patterns of symptoms caused by using a substance that an individual continues taking despite its negative effects. There are 11 diagnostic criteria fall under four basic categories — impaired control, physical dependence, social problems and risky use: using more of a substance than intended or using it for longer than you’re meant to; trying to cut down or stop using the substance but being unable to; experiencing intense cravings or urges to use the substance; needing more of the substance to get the desired effect; developing withdrawal symptoms when not using the substance; spending more time getting and using drugs and recovering from substance use; neglecting responsibilities at home, work or school because of substance use; continuing to use even when it causes relationship problems; giving up important or desirable social and recreational activities due to substance use; using substances in risky settings that put you in danger; continuing to use despite the substance causing problems to your physical and mental health
Problematic use of alcohol	1	Persistent and problematic use of alcohol despite significant distress and impairment, with associated behavioral and physical symptoms, including withdrawal, tolerance, and cravings. Alcohol use disorder is characterized by failure to fullfill daily responsibilites and role obligations, alcohol-seeking behavior, unsuccesful efforts to control alcohol use, drinking despite potential hazards (e.g., drinking while driving), the need for increased amounts of alcohol to achieve its effects (tolerance), and withdrawal symptoms when one stops or reduces alcohol intake (e.g., hand tremors, nausea, agitation, hallucinations)
Autism spectrum disorder	1	DSM-V recognizes two main symptom areas: deficits in social communication and interaction AND restricted, repetitive behaviors, interests, or activities. These symptoms appear early in a child’s development—although diagnosis may occur later. Autism is diagnosed when symptoms cause developmental challenges that are not better explained by other conditions
Self-harm	1	Deliberate, self-inflicted destruction of body tissue without suicidal intent and for purposes not socially sanctioned, includes behaviors such as cutting, burning, biting and scratching skin, especially present during adolescence
Adjustment disorder	1	Presence of emotional or behavioral symptoms in response to an identifiable stressor(s) occurring within 3 months of the onset of the stressor(s). It could be present with anxiety, depressed mood or both, or disturbance of conduct

To exclude a possible influence of psychopharmacological active ingredients on the expression of BDNF and GDNF, we excluded subjects treated with antidepressant and antipsychotic drugs or positive for narcotic substances. Three of the twenty subjects studied regularly took only a mild therapy with sedative drugs (benzodiazepines), whose active ingredients, based on the results of toxicological analyses, still fell within the therapeutic prescribed dosage range.

### 2.2 Methods

For each case, the entire brain was removed during the autopsy examination, with appropriate preservation in formol solution for 3 weeks to perform a correct fixation of the tissues. The brain was dissected using sagittal cuts, identifying and taking four areas of interest from the right hemisphere: the prefrontal cortex, cingulate gyrus, basal nuclei, and hippocampus.

All the samples obtained were embedded in paraffin and treated by immunohistochemical staining for anti-BDNF and anti-GDNF antibodies to proceed with the search for neurotrophins. The two antibodies used are BDNF (monoclonal mouse antibody Catalog Number: 66292-1-ig, Proteintech Group, Chicago, United States), and GDNF (monoclonal rabbit antibody, Catalog Number: orb572592, Biorbyt Ltd., Cambridge, United Kingdom). For both antibodies, it was used the Protein Atlas website for the selection of positive and negative controls, and then it was performed various tests for the individuation of the correct pretreatment and dilution, according to the indications of the Production Company, published papers, and the suggestions of www.atlasantibodies.com.

For each case, sections of approximately 4 μm were made on the microtome. The sections, mounted on a slide, were hydrated in decreasing alcohol solutions. After inhibiting endogenous peroxidase, the sections were subjected to antigen retrieval in Citric acid 0.1 M and subsequently incubated with the primary antibody (dilution 1:200 for BDNF and 1:500 for GDNF). The formation of the immune complex was highlighted by applying a Streptavidin-Biotin system (HRP-DAB system research and development kit CTS005, R&D Systems, Inc., Minneapolis, MN, United States). The reaction was visualized by peroxidation of 3,3′-diaminobenzidine (DAB). Once the reaction was verified, the nuclei were counter-stained with hematoxylin, and subsequently slides were subjected to dehydration. The slide was then mounted and observed using a Nikon Eclipse E90i optical microscope, using the NIS – Elements F program. BDNF and GDNF immunopositivity in the right frontal cortex, hippocampus (dentate gyrus (DG) -Lacunar-molecular, Radiate, Granular layers and Hilus:.), girus of the cingulum, and basal nuclei. For the hippocampus, the granular layer and hilus are the selected areas for the statistical analysis because they are more significant. Four visual fields of approximately 350 μm × 350 μm were randomly selected in interest of each area for each sample of cases and controls. Images for each slide were acquired with a digital camera (Nikon) connected to the microscope at 10x for an exhaustive semiquantitative, preliminary evaluation of the reaction and after at 40 × magnification in the more significant area for quantification. The parameters for image acquisition were established at the beginning of the observation and kept constant for all images. Quantifying cells positive for DAB staining was performed using ImageJ software (imagej.nih.gov/ij/) and expressed as the number of positively stained cells per analyzed area. Quantifications were expressed as the number of positive-stained cells/analyzed area. For hippocampus we quantized the radiate layer and hilus because are the areas. Blind researchers performed histological analyses concerning the information about the cases. The blinding of the data was maintained until the analysis was terminated.

### 2.3 Statistic analysis

Data were analyzed using Windows GraphPad Prism 10 software for Windows (La Jolla, CA, United States). The data were analyzed through the two-way ANOVA analytical system (two-way ANOVA) for two independent factors. A *P*-value < 0.05 was considered statistically significant. Results are expressed as means ± standard deviation.

## 3 Results

### 3.1 Prefrontal cortex

**FIGURE 1:**
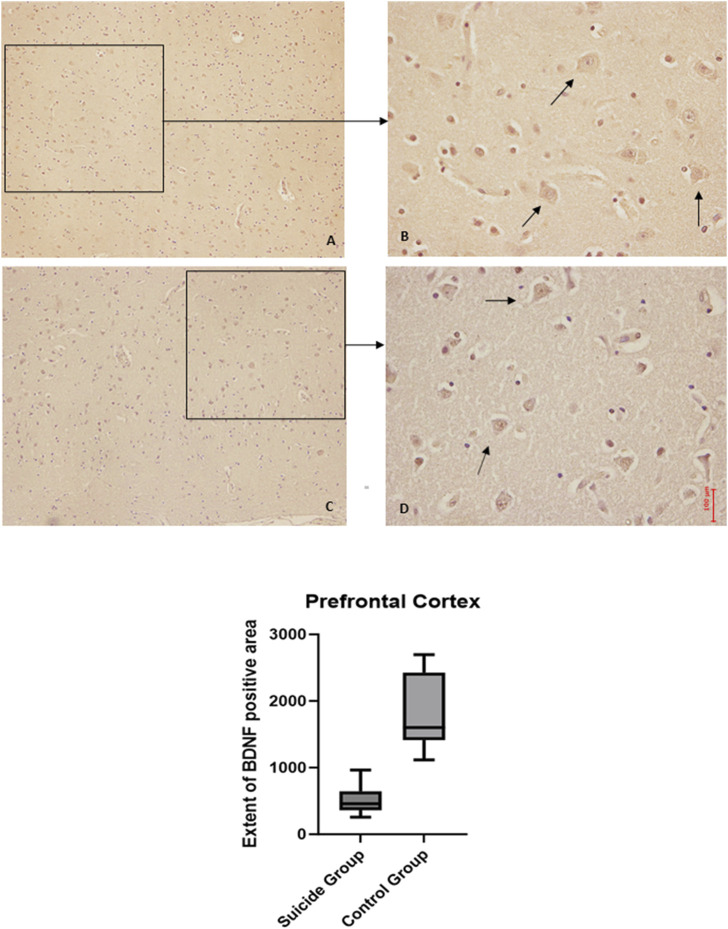
BDNF immunohistochemical expression in the prefrontal cortex. Prefrontal cortex immunohistochemistry results: normal immunohistochemistry reaction of BDNF in the control case, see arrows **(A)** 10x magnification **(B)** 40x magnification; reduced expression of BDNF in a sample of suicide cases, see arrows **(C)** 10x magnification **(D)** 40x magnification. In the lower part of the figures, the graphical representation of the statistical analysis, the percentage decrease of BDNF in the suicide cases group is 24%.

**FIGURE 2:**
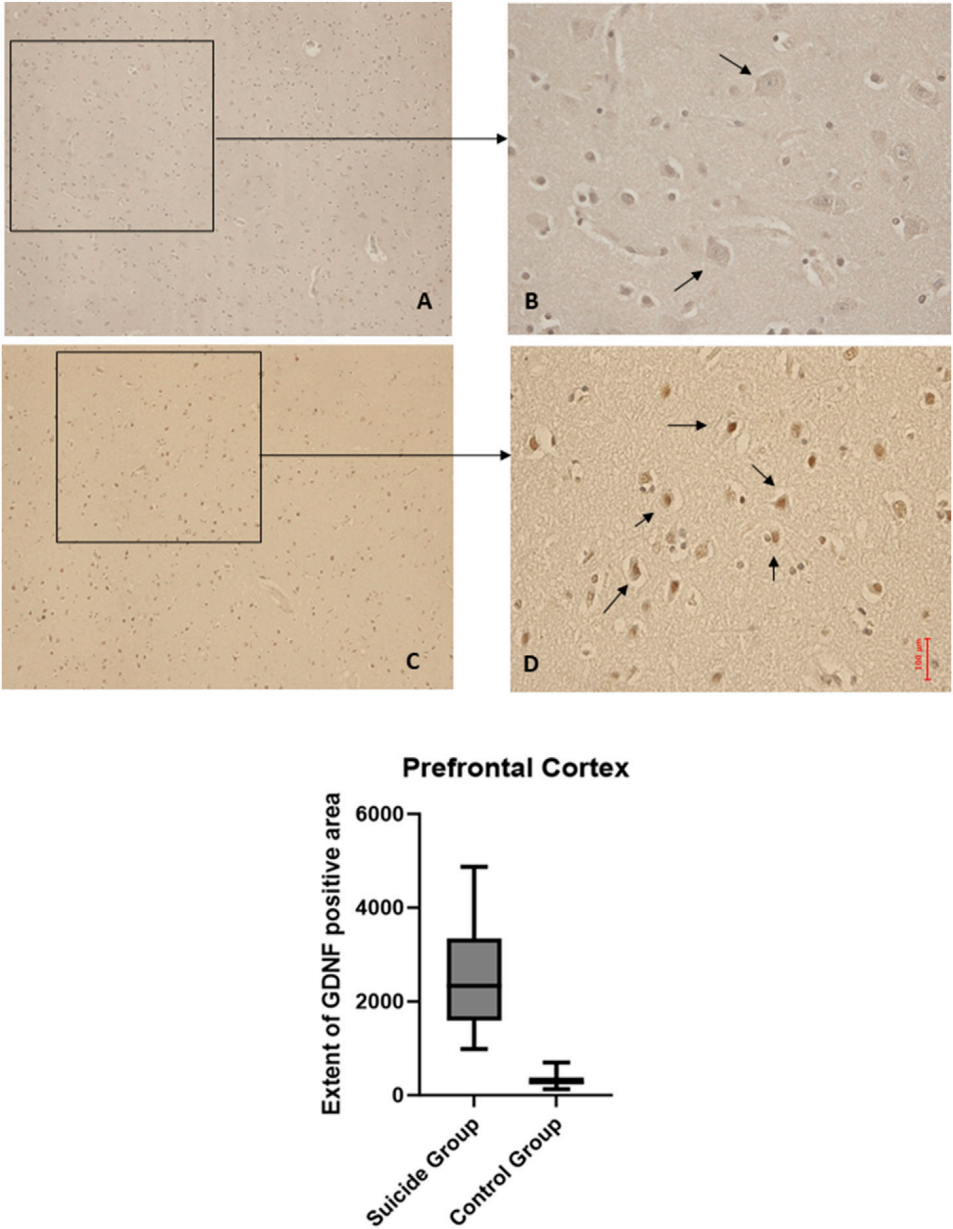
GDNF immunohistochemical expression in the prefrontal cortex. Prefrontal cortex immunohistochemistry results: basal immunohistochemistry reaction of GDNF in thecontrol case (the control case used is the same as Figure 1, you can see in consecutive slice section the difference of reaction between the two markers), see arrows **(A)** 10x magnification **(B)** 40x magnification; over-expression of GDNF in a sample of suicide case, see arrows **(C)** 10x magnification **(D)** 40x magnification. The graphical representation of the statistical analysis is in the lower part of the figures.

The two-way ANOVA highlighted a statistically significant difference in both cases (*p* < 0.0001). In particular, a reduced expression of BDNF was noted in suicide cases compared to controls, while the expression of GDNF was increased in cases compared to controls (Figures 1, 2).

### 3.2 Hippocampus

**FIGURE 3:**
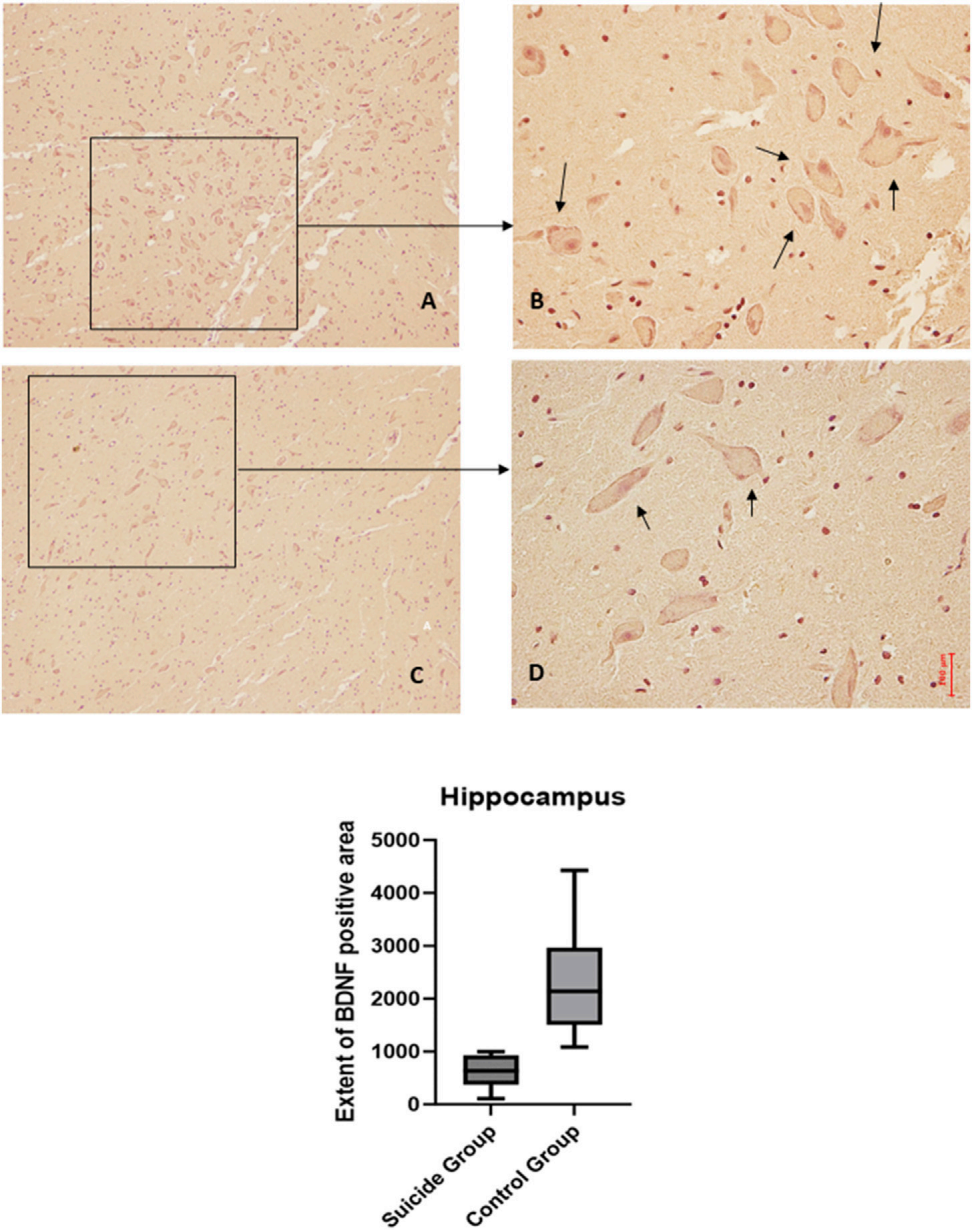
BDNF immunohistochemical expression in the hippocampus. Hippocampus (dentate gyrus) immunohistochemistry results: normal immunohistochemistry reaction of BDNF in the control case (granular layer of dentate gyrus), see arrows **(A)** 10x magnification **(B)** 40x magnification; reduced expression of BDNF in a sample of suicide case (granular layer of dentate gyrus), see arrows **(C)** 10x magnification **(D)** 40x magnification. In the lower part of the figures, the graphical representation of the statistical analysis, the percentage decrease of BDNF in the suicide cases group is 27%.

**FIGURE 4:**
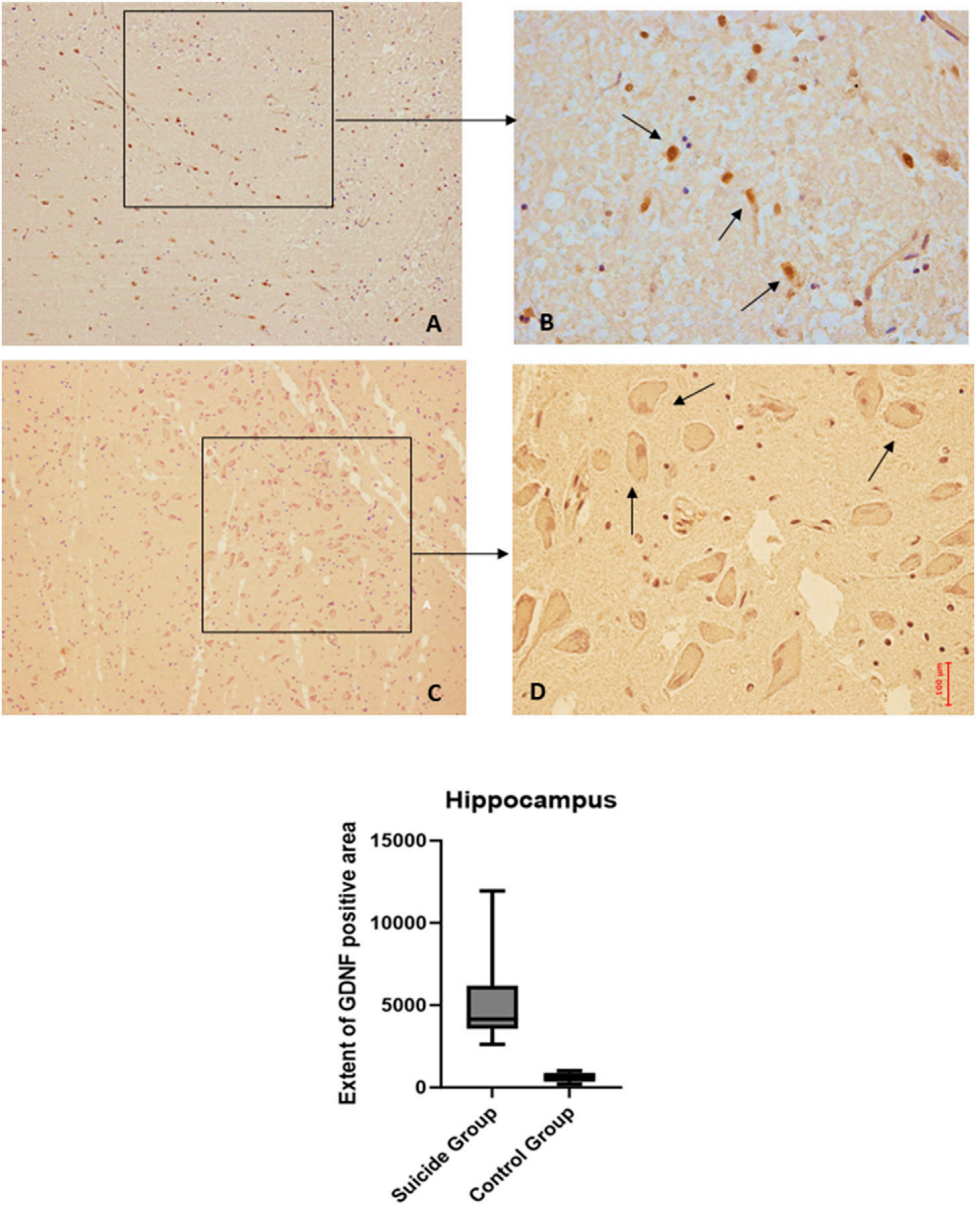
GDNF immunohistochemical expression in the hippocampus. Hippocampus (dentate gyrus) immunohistochemistry results: basal immunohistochemistry reaction of GDNF in the control case (hilus of dentate gyrus), see arrows **(A)** 10x magnification **(B)** 40x magnification; over-expression of GDNF in a sample of suicide case (granular layer of dentate gyrus), see arrows **(C)** 10x magnification **(D)** 40x magnification. In the lower part of the figures is the graphical representation of the statistical analysis.

The application of the two-way ANOVA test returned a statistically significant difference between the expression of BDNF (*p* < 0.001) and GDNF (<0.0001). In particular, it was possible to highlight a reduced expression of BDNF in suicide cases compared to controls, while the expression of GDNF was found to be increased in cases compared to controls (Figures 3, 4).

### 3.3 Gyrus of the cingulum

**FIGURE 5:**
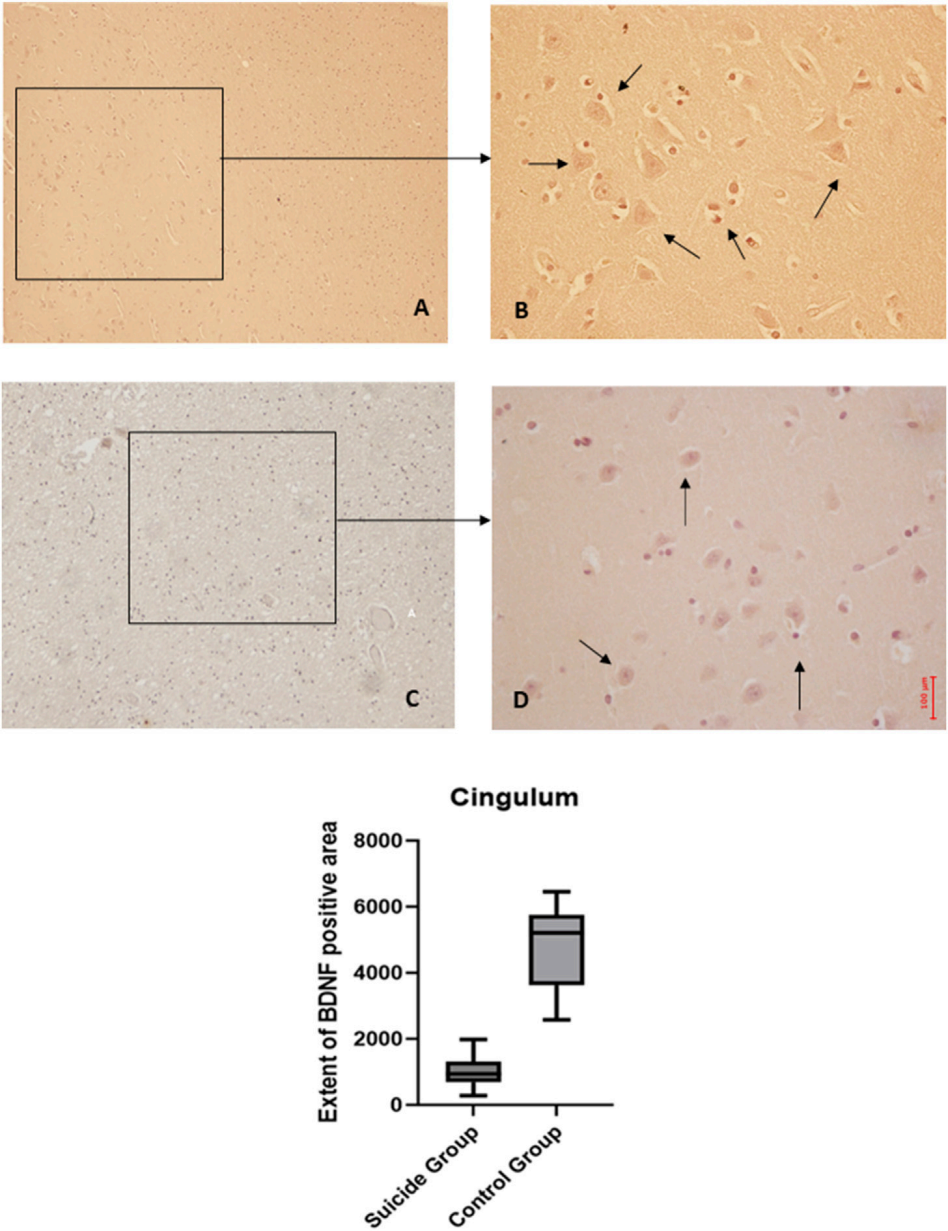
BDNF immunohistochemical expression in cingulum. Cingulum immunohistochemistry results: normal immunohistochemistry reaction of BDNF in the control case, see arrows **(A)** 10x magnification **(B)** 40x magnification; reduced expression of BDNF in a sample of suicide cases, see arrows **(C)** 10x magnification **(D)** 40x magnification. In the lower part of the figures, the graphical representation of the statistical analysis shows that the percentage decrease of BDNF in the suicide cases group is 21%.

**FIGURE 6:**
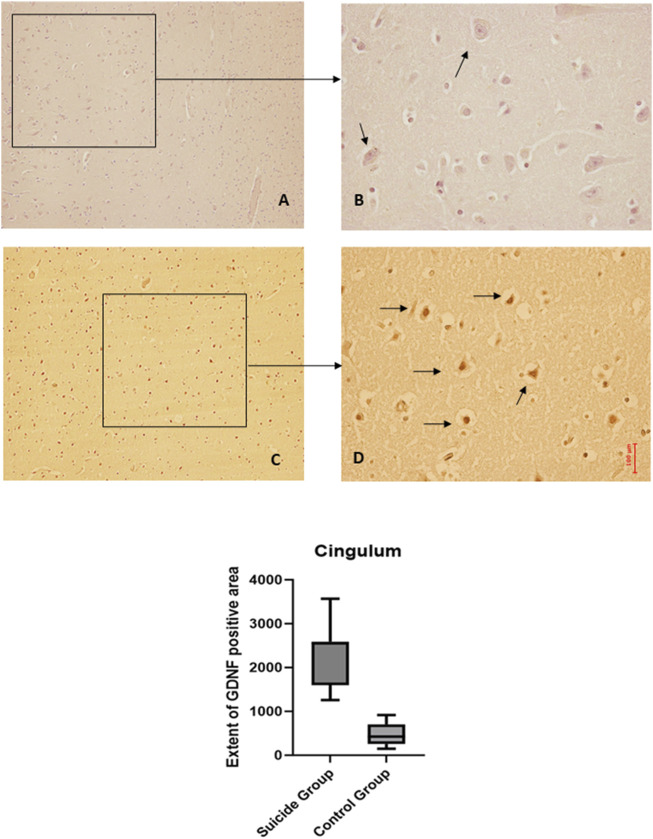
GDNF immunohistochemical expression in cingulum. Cingulum immunohistochemistry results: basal immunohistochemistry reaction of GDNF in the control case, see arrows **(A)** 10x magnification **(B)** 40x magnification; over-expression of GDNF in a sample of suicide case, see arrows **(C)** 10x magnification **(D)** 40x magnification. The graphical representation of the statistical analysis is in the lower part of the figures.

The two-way ANOVA analysis highlighted a statistically significant difference in both cases (*p* < 0.0001). In particular, a reduced expression of BDNF was noted in suicide cases compared to controls, although less marked than in the other areas examined. Also, in this case, the expression of GDNF is increased in cases compared to controls (Figures 5, 6).

### 3.4 Basal nuclei

**FIGURE 7:**
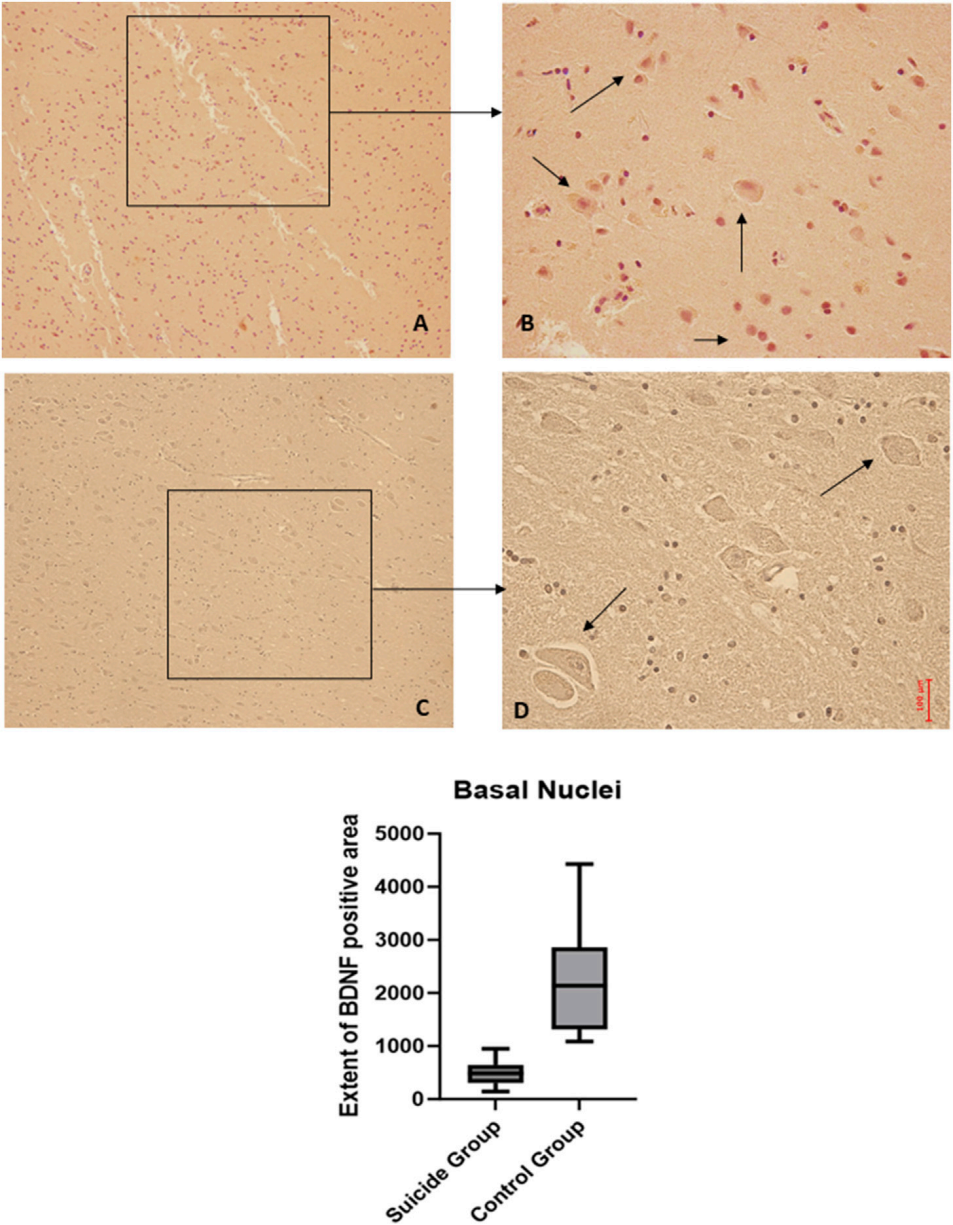
BDNF immunohistochemical expression in basal nuclei. Basal nuclei immunohistochemistry results: normal immunohistochemistry reaction of BDNF in the control case, see arrows **(A)** 10x magnification **(B)** 40x magnification; reduced expression of BDNF in a sample of suicide case, see arrows **(C)** 10x magnification **(D)** 40x magnification. In the lower part of the figures, the graphical representation of the statistical analysis shows that the percentage decrease of BDNF in the suicide cases group is 21%.

**FIGURE 8:**
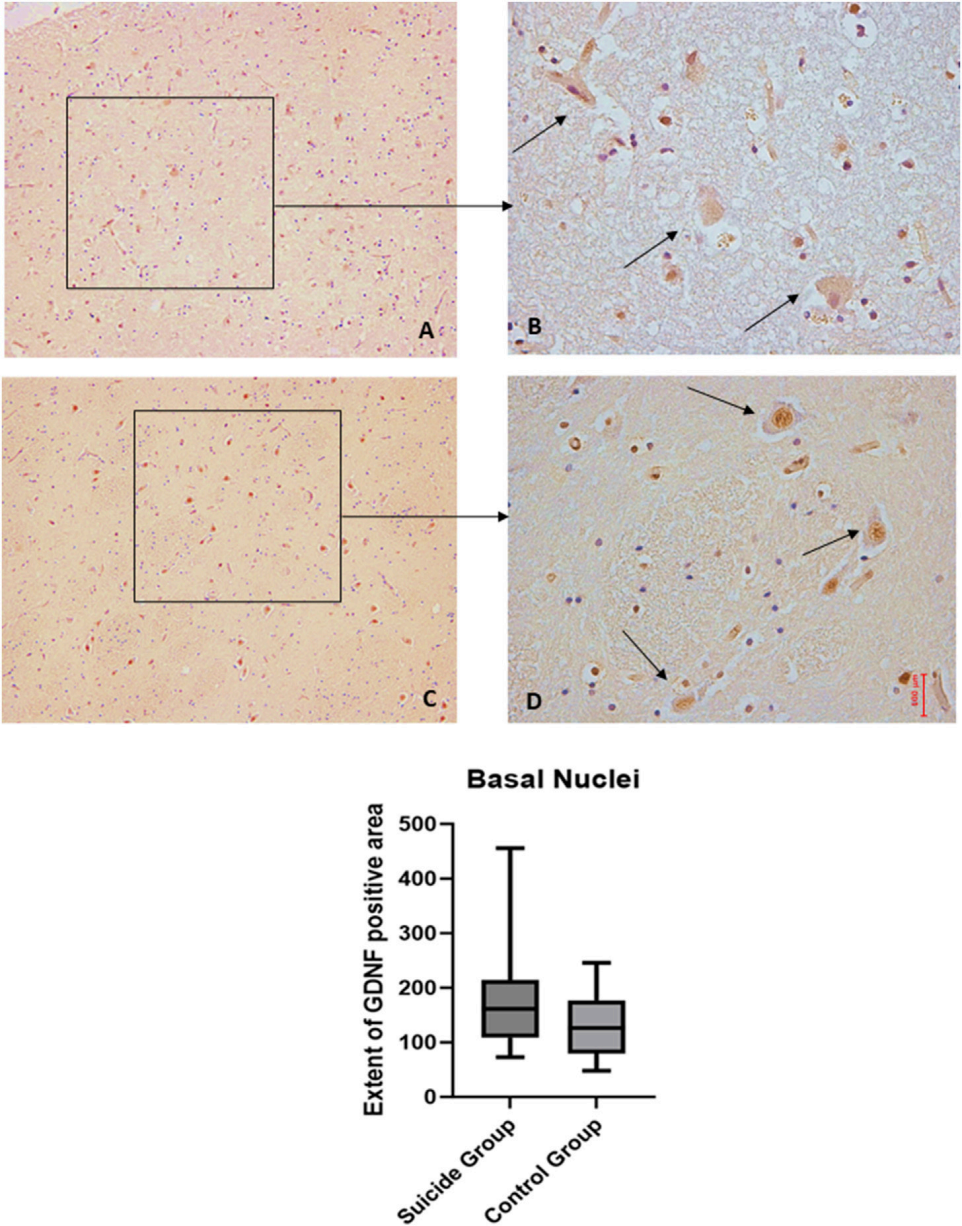
GDNF immunohistochemical expression in basal nuclei. Basal nuclei immunohistochemistry results: basal immunohistochemistry reaction of GDNF in the control case, see arrows **(A)** 10x magnification **(B)** 40x magnification; similar basal expression of GDNF in a sample of suicide case, see arrows **(C)** 10x magnification **(D)** 40x magnification. In the lower part of the figures is the graphical representation of the statistical analysis.

The two-way ANOVA showed a statistically significant difference in the expression of BDNF (*p* < 0.001), while no statistically significant differences were highlighted in GDNF (*p* < 0.1). In particular, a reduced expression of BDNF was noted in suicide cases compared to controls, while a minimal increase in GDNF expression was observed in cases compared to controls (Figures 7, 8).

## 4 Discussion

There are few studies in the literature relating to the measure of BDNF and GDNF on post-mortem samples of suicidal subjects, especially those without psychiatric pathologies. Our work aims to contribute to science to identify molecules useful as markers of suicide risk. These neurotrophins could also be potential pharmacological targets for researching and developing new targeted therapies.

The sample examined in our study was composed of twenty subjects who died by suicide and ten controls who died of non-suicidal causes, matched for sex and age, who underwent autopsy examination. 90% of subjects were male (18 of 20), 10% were female (2 of 20), 13 of them (65%) were under 50 years old, and 7 (35%) were over 50 years old. Furthermore, 6 out of 20 individuals (30%) did not have any diagnosis of psychiatric disorder. However, they had been involved in recent stressful events, for example, bereavement, job loss, and imprisonment. The remaining 14 cases (70%), however, presented a psychiatric pathology; of these, six were affected by mood disorders (five from major depressive disorder, one from bipolar disorder), eight from other types of diagnosed psychiatric pathology (pathological gambling, substance use disorder, problematic use of alcohol, autism spectrum disorder, self-harm, adjustment disorder). Five of the 20 cases analyzed (25%) were subjects subjected to a prison regime, another risk factor.

To exclude the possible influence of psychopharmacological active ingredients on the expression of BDNF and GDNF, the authors excluded patients on therapy with antidepressant and antipsychotic drugs and those who tested positive for narcotic and psychoactive substances in the toxicological analyses. Therefore, subjects who committed suicide due to overdose were also excluded. Only three of the 20 subjects studied were taking mild therapy based on non-neuroleptic or antidepressant drugs (mainly benzodiazepines) whose active ingredients, based on the results of toxicological analyses, were still within the therapeutic dosage range.

11 out of 20 subjects (55%) died from hanging [the most used method, according to literature data (Baldari et al., 2021)], 4 out of 20 (20%) from precipitation, 2 out of 20 (10%) from stab wounds, 2 in 20 (10%) by drowning and 1 in 20 (5%) by gunshot wounds.

The results of our work, which used an immunohistochemical method widely used in the field of forensic pathology (Baldari et al., 2021), highlighted a statistically significant reduction in BDNF levels in the various brain regions examined in all twenty suicidal subjects compared to the ten controls. In particular, the areas most affected are the prefrontal cortex and cingulate gyrus (*p* < 0.0001), to a slightly lesser extent, hippocampus and basal nuclei (*p* < 0.001). These data agree with the literature (Middlemas et al., 1991; Karege et al., 2002; Dwivedi et al., 2003; Karege et al., 2005b; Tripp et al., 2012), which reports significant decreases in BDNF levels in the prefrontal cortex, hippocampus, and cingulate gyrus. Furthermore, it is interesting to consider that the global examination of the different brain areas highlighted a statistically significant reduction in BDNF in suicidal subjects compared to controls (*p* < 0.0001).

The encephalic expression of GDNF was increased in all 20 study subjects. In particular, the authors observed a statistically significant (*p* < 0.0001) increase in GDNF in suicidal subjects compared to controls at the prefrontal cortex, hippocampus, and cingulate gyrus. In the basal nuclei of suicidal subjects, however, there was a slight increase in GDNF levels compared to controls, which was not statistically significant. Interestingly, the global examination of the different brain areas highlighted an increase in GDNF in suicidal subjects, compared to controls, which was statistically significant (*p* < 0.0001).

Our research is unique, as we analyzed the expression of GDNF on the brain tissue of suicidal subjects without therapy. The few studies in the literature on GDNF have evaluated its concentration in the peripheral blood of patients with depressive or, more generally, mood disorders.

The results obtained agree with the data of another work (Rosa et al., 2006), which documented an increase in GDNF in the peripheral blood of patients who have bipolar disorder in the depressive phase. Thus, increased BDNF synthesis could be a characteristic of acute episodes of mood disorders, although the literature is unclear (Takebayashi et al., 2006; Otsuki et al., 2008).

An important point of the study is that we have only selected autoptic cases of subjects who have not undergone drug therapy. In literature, most of the studies concern subjects on antidepressant drug therapy, finding a reduction in the expression of GDNF in plasma or blood (Maheu et al., 2015). On the contrary, in our study, the GDNF molecule in the brain is increased. It suggests that central GDNF signaling may represent a novel target for antidepressant treatment (Maheu et al., 2015).

In our study, we evaluate neurotrophic expression at the brain tissue, while other studies evaluate it at the plasma peripheral level (Sun et al., 2019). It would be interesting to conduct a multidisciplinary clinic-based assessment to observe whether the reduction in GDNF correlates with an improvement in symptoms.

The authors analyzed three further subgroups of the 20 cases: six subjects suffering from psychiatric pathology, eight subjects suffering from mood disorders, and six normal subjects. This analysis revealed a statistically significant reduction (*p* < 0.0001) in the expression of BDNF in all psychiatric subjects compared to the control group in all encephalic areas. In contrast, no statistically significant variations appeared in comparison between depressed individuals and those with other mental disorders. The same result was obtained with the GNDF, with a statistically significant increase (*p* < 0.0001) in all brain areas of psychiatric subjects compared to the control group. In contrast, there were no statistically significant variations in the comparison between depressed individuals and those with other psychic disorders.

## 5 Conclusions

The results we obtained on the expression of BDNF and GDNF, in agreement with data from works published in the literature, support the hypothesis that lower levels of BDNF and higher levels of GDNF correlate with an increased risk of suicide. The use of these markers could have several clinical implications. BDNF and GDNF testing could be valuable markers for screening patients most at risk of suicide based on demographic and clinical data to estimate suicidal risk. These markers measured *in vivo* and correlated with clinical symptomatology could help assess the severity of depressive symptoms, such as the presence of polymorphism of the BDNF gene (Val66Met). Further studies could have important clinical implications using BDNF and GDNF as targets for specific pharmacological therapies for depression.

This study could be a pilot study to continue the research on a larger sample of subjects who died as a result of suicide. It would, therefore, be necessary to increase the sample size, standardize the data collection methods, and group the samples according to their characteristics. In our study, there is a wide heterogeneity regarding the method of suicide and psychiatric pathology. In addition, the exclusion of psychoactive substance users may have led to a selection bias, although it was necessary to identify the modification in BDNF and GDNF correctly.

## Data Availability

The raw data supporting the conclusions of this article will be made available by the authors, without undue reservation.
